# Supplementary material for: Serological test performance for bovine tuberculosis in cattle from herds with evidence of on-going infection in Northern Ireland
Source: PLoS One. 2021 Apr 13;16(4):e0245655. doi: 10.1371/journal.pone.0245655 (PMC8043403; doi:10.1371/journal.pone.0245655)
Supplement: Attachment — Submitted filename: Response_to_reviewers.docx [file pone.0245655.s003.docx]

**Response to academic editor and reviewers**

**Journal Requirements:**

When submitting your revision, we need you to address these additional requirements.

**1. Please ensure that your manuscript meets PLOS ONE's style requirements, including those for file naming. The PLOS ONE style templates can be found at**

<https://journals.plos.org/plosone/s/file?id=wjVg/PLOSOne_formatting_sample_main_body.pdf> and

<https://journals.plos.org/plosone/s/file?id=ba62/PLOSOne_formatting_sample_title_authors_affiliations.pdf>

 The revised manuscript should now meet the requirements as set out in the links above.

**2. In your Methods section, please provide the name of the slaughterhouse where the animals were sacrificed.**

Name of slaughterhouse (WD Meats Ltd.) provided – line 108 in Manuscript file.

**3. Please include captions for your Supporting Information files at the end of your manuscript, and update any in-text citations to match accordingly. Please see our Supporting Information guidelines for more information:** [http://journals.plos.org/plosone/s/supporting-information](about:blank).

Title and caption added at the end of the manuscript.

**Reviewer 1 comments**

**Line 126: pokeweed mitogen is the internal positive control, right? I suggest you mention it.**

Yes, pokeweed mitogen is the positive control. Text added - line 131 in Manuscript file.

**Line 186-188: you mention here the collection of a selection of tissues for histological examination, can you present the results of this examination in the manuscript? If it's not possible, then there is no need to mention the tissue sample collection in formalin for histology.**

Text relating to collection of tissues for histology removed from this section of the manuscript.

**Reviewer 2 comments**

**Abstract: purpose of the study? Case study**

The case study presents information on the use of one of the serology tests (IDEXX M. bovis Ab test) in a Northern Ireland herd experiencing repeated TB breakdowns. The Department of Agriculture, Environment and Rural Affairs permitted exceptional use of this serology test in this herd in an attempt to detect additional positive animals (possibly anergic animals un responsive to SICCT or IFNƔ) previously undetected by SICCT. These details have not been included in the abstract but outlined in the materials and methods under the section titled ‘Case study herd’.

**Lines 51-52: how is confounding effect used here?**

Confounding effect is used here in terms of the effect of concurrent infections such as Johne’s disease and liver fluke on diagnostic tests. Text added - line 56 in Manuscript file.

**Line 84: bovine TB problem – how was this defined?**

“Bovine TB problem” removed. The text now reads as follows: “Samples intended for analysis were taken from cattle from Northern Ireland herds eligible for inclusion in the IFNƔ testing scheme operated by the Department for Agriculture, Environment and Rural Affairs (DAERA), Northern Ireland.” Lines 89-91 in Manuscript file.

**Lines 92-93: highlighted in PDF but no comment**

No changes made

**Line 170: highlighted but no comment**

No changes made

**Lines 192-194: move to discussion**

This sentence describes the AUC, and its interpretation. It should remain in the methods section. Lines 196-198 in Manuscript file.

**Line 195: change was to were**

Changed as requested.

**Lines 197-198: post mortem results – microbiological testing / culture?**

Post mortem status includes abattoir findings (lesioned / not lesioned) and microbiological confirmation. Text added – lines 207-208 in Manuscript file.

**Lines 206-207: What was the outcome of these logistic regression models? How results are interpreted? Odd ratios.... for what?**

The outcome of the models is the binary test result [‘positive’ 1; ‘negative’ 0] for each test – text added in lines 218-219.

Odds ratios removed as they are not discussed in the text.

**Lines 210-211: sex, age, breed – add into study objectives**

Text added to study objectives. Lines 81-82 in Manuscript file.

**Line 215: consider modifying title to keep consistency with previous mentions of this part of this manuscript. "Case herd study", perhaps.**

Title changed to ‘Case herd study’. Line 226 in Manuscript file.

**Lines 222-223: How many of these disclosed lesions at slaughter?**

Ten out of 148 positive animals were confirmed at post-mortem inspection or in the laboratory (histology or bacteriology). Lines 236-237 in Manuscript file.

**Lines 226-227: This is confusing. What do you mean 'in contact', if these animals already belonged to this herd? In contact with what?**

‘In contact’ means animals in contact, housed or managed alongside TB reactor cattle in the herd. Lines 239-240 in Manuscript file.

**Lines 228-231: Consider moving to discussion section.**

Text should stay in materials and methods under section titled ‘Case herd study’. Lines 244-246 in Manuscript file.

**Lines 250-251: There was significant (p<0.001) moderate agreement between the serological tests ranging from a kappa of 0.40 (IDEXX and Enfer 4ag) to 0.55 (Enfer 2ag and Enfer 4ag). This comparison is not stated in the objective/purpose statement. Either add to it, or delete these results.**

Comparison deleted from manuscript.

**Line 293: Age, sex, breed associations….Were these stated in the objectives?**

Text added to objectives. Lines 81-82 in Manuscript file.

**Line 296: What models were used for evaluating these associations?**

Odds ratios (ORs) are from logistic regression models.

**Line 303: Do all these results come from standard 2x2 tables?**

Yes, Chi square tests are univariable tests (i.e. 2x2 tests).

**Line 310: amend title**

Amended to ‘Case herd study’ as suggested above.

**Lines 311-314: move to materials and methods**

Moved as requested. Lines 244-245 in Manuscript file.

**Lines 324-327: Is this animal part of the 9 animals indicated above?**

No this animal was not one of the 9 animals indicated above. This animal was slaughtered at a later stage, some months after the 9 animals were surrendered on the basis of the serological investigation.

**Line 356: ‘see’ not needed**

Removed.

**Line 360: Infected..... based on what criteria? Serological test result?**

Confirmed infected animals – based on post-mortem findings, histology and or bacteriology. Lines 408-409 in Manuscript file.

**Line 375: M. bovis – should be in italics**

M. bovis italicised. Line 423 in Manuscript file.

**Line 380: ‘see’ not needed**

Removed.

**Line 395: from the prospective study results……. are you referring here to the other results (study) on this manuscript? I suggest to keep language consistency to ease the read**

Yes this text is referring to the other results in this manuscript. Text now reads as: ‘This field application of the test in a particularly problematic herd appears to corroborate our other findings presented in this manuscript.’ Lines 440-442.

**Table 1: mean not appropriate for analyses conducted. PPV /NPV need explanation / interpretation in the text**

Mean removed from table 1 and other tables.

Explanation for PPV/NPV added to text. Lines 200-204.

**Table 2: Odds ratio - refer to these results on the text. Prev (comparator) and test prevalence – explain, footnote.**

Odds ratio removed from table 2. Prev and test prevalence explained in table 1 footnote.

**Table 3: Comparator – criteria? Odds ratio - refer to these results on the text. Mean not appropriate**

Odds ratio and mean removed from table 3.

**Table 4: Odds ratio / P value – needed?**

Removed from table 4.

**Table 5: change ‘and’ to ‘by’. Numbers italicised represent ante-mortem negative animals that were serologically test positive - This explanation should not be in the title.**

‘And’ changed to ‘by’.

Explanation now in table footnote.
